# Seasonality of acquisition of respiratory bacterial pathogens in young children with cystic fibrosis

**DOI:** 10.1186/s12879-017-2511-9

**Published:** 2017-06-09

**Authors:** Kevin J. Psoter, Anneclaire J. De Roos, Jon Wakefield, Jonathan D. Mayer, Margaret Rosenfeld

**Affiliations:** 1 Department of Pediatrics, School of Medicine, The Johns Hopkins University Bayview Medical Center, 5200 Eastern Ave, Mason F. Lord Bldg, Suite 4200, Baltimore, MD 21224 USA; 2 Department of Environmental and Occupational Health, Drexel University School of Public Health, Philadelphia, PA USA; 3 Departments of Biostatistics and Statistics, University of Washington, Seattle, WA USA; 4 Departments of Epidemiology, Geography, Global Health, Medicine (Allergy and Infectious Diseases), Family Medicine, and Health Services, University of Washington, Seattle, WA USA; 5 Division of Pulmonary Medicine, University of Washington School of Medicine, Seattle, WA USA; 6 Department of Pediatrics, Seattle Children’s Hospital, Seattle, WA USA

**Keywords:** Cystic fibrosis, Seasonality, MRSA, *Stenotrophomonas maltophilia*, *Achromobacter xylosoxidans*, *Haemophilus influenzae*

## Abstract

**Background:**

Seasonal variations are often observed for respiratory tract infections; however, limited information is available regarding seasonal patterns of acquisition of common cystic fibrosis (CF)-related respiratory pathogens. We previously reported differential seasonal acquisition of *Pseudomonas aeruginosa* in young children with CF and no such variation for methicillin-susceptible *Staphylococcus aureus* acquisition. The purpose of this study was to describe and compare the seasonal incidence of acquisition of other respiratory bacterial pathogens in young children with CF.

**Methods:**

We conducted a retrospective study to describe and compare the seasonal incidence of methicillin-resistant *Staphylococcus aureus* (MRSA), *Stenotrophomonas maltophilia*, *Achromobacter xylosoxidans*, and *Haemophilus influenzae* acquisition in young CF patients residing in the U.S. using the Cystic Fibrosis Foundation National Patient Registry, 2003-2009. Log-linear overdispersed Poisson regression was used to evaluate seasonal acquisition of each of these pathogens.

**Results:**

A total of 4552 children met inclusion criteria. During follow-up 910 (20%), 1161 (26%), 228 (5%), and 2148 (47%) children acquired MRSA, *S. maltophilia*, *A. xylosoxidans* and *H. influenzae,* respectively. Compared to winter season, MRSA was less frequently acquired in spring (Incidence Rate Ratio [IRR]: 0.79; 95% Confidence Interval [CI]: 0.65, 0.96) and summer (IRR: 0.69; 95% CI: 0.57, 0.84) seasons. Similarly, a lower rate of *A. xylosoxidans* acquisition was observed in spring (IRR: 0.59; 95% CI: 0.39, 0.89). For *H. influenzae*, summer (IRR: 0.88; 95% CI: 0.78, 0.99) and autumn (IRR: 0.78; 95% CI: 0.69, 0.88) seasons were associated with lower acquisition rates compared to winter. No seasonal variation was observed for *S. maltophilia* acquisition.

**Conclusion:**

Acquisition of CF-related respiratory pathogens displays seasonal variation in young children with CF, with the highest rate of acquisition for most pathogens occurring in the winter. Investigation of factors underlying these observed associations may contribute to our understanding of the aetiology of these infections and guide future infection control strategies.

**Electronic supplementary material:**

The online version of this article (doi:10.1186/s12879-017-2511-9) contains supplementary material, which is available to authorized users.

## Background

Cystic fibrosis (CF), an autosomal recessive disorder, is characterized by chronic and recurring endobronchial infection, resulting in progressive structural lung disease and, generally, premature death. Beginning early in life, abnormal mucociliary clearance promotes colonization by a variety of bacteria [1, 2]. *Haemophilus influenzae* and methicillin-susceptible *Staphylococcus aureus* (MSSA) are frequently among the first organisms isolated from respiratory cultures [1]; however, their effect on clinical outcomes is debated [2]. *Pseudomonas aeruginosa,* a Gram-negative bacterium frequently first isolated from respiratory cultures in early childhood, is regarded as the most important CF-related respiratory pathogen [3, 4]. Other gram-negative organisms and methicillin-resistant *S. aureus* (MRSA) often follow, and appear to also adversely affect clinical outcomes [2, 5].

While risk factors for specific bacterial infections have been reviewed [2, 5, 6], little information is available regarding the incidence patterns of these infections. *P. aeruginosa* appears to be typically initially acquired from the natural environment; however, the sources for other organisms are far less clear. A more complete understanding of the incidence patterns of acquisition of other pathogens may provide an opportunity for the identification of potential aetiologic factors, as well as refinement of infection control strategies for these infections. Seasonal variation of infectious diseases is often observed [7] and increasing evidence suggests that many Gram-negative infections display such patterns [8, 9]. Previously, we reported differential seasonal acquisition of initial *P. aeruginosa* in young children with CF and no such variation for MSSA infections [10]. The purpose of this study was to describe and compare seasonal incidence for other common CF-related respiratory bacterial pathogens including MRSA, *Stenotrophomonas maltophilia*, *Achromobacter xylosoxidans* and *Haemophilus influenzae*, in young CF patients residing in the U.S.

## Methods

### Study population

We performed a retrospective cohort study of the seasonal incidence of MRSA, *S*. *maltophilia*, *A*. *xylosoxidans*, and *H. influenzae* acquisition in U.S. children with CF <6 years of age using the Cystic Fibrosis Foundation National Patient Registry, 2003-2009. The Registry contains detailed information on patient demographic and disease characteristics, including results of respiratory cultures at each clinical encounter [11]. Current clinical recommendations are for quarterly cultures (i.e. four times per year) [12]. Children <6 years of age typically do not expectorate sputum so the primary source of cultures in this age range is oropharyngeal swabs. Culturing for the infectious agents, and in the case of MRSA, antimicrobial resistance, is performed at the individual clinical microbiology laboratories and based on the selective media recommendations for each pathogen by the Cystic Fibrosis Foundation [13].

The study population consisted of children born after December 31, 2002. Only those children who: 1) were diagnosed with CF and 2) had a first respiratory culture performed prior to two years of age were included in the study cohort. This study was approved by the institutional review board at the University of Washington and the Cystic Fibrosis Foundation Registry Committee.

### Statistical analysis

For each organism, demographic and disease characteristics were compared between those patients acquiring and remaining pathogen-free during follow-up using the Student *t* test with unequal variances and the Chi square test for continuous and categorical variables, respectively.

Seasons were defined as: winter (December-February months), spring (March-May), summer (June-August) and autumn (September-November). Seasonal incidence for each of the respiratory pathogens was calculated over the six-year study period. The incident culture for each organism was defined as the first respiratory culture from which that organism was isolated as recorded in the Registry. Numerator data included the number of cases occurring during each season and the denominator was taken as the total person-seasons of children at risk for initial acquisition. Children entered the study upon first clinical encounter recorded in the Registry and remained at risk until either the pathogen was cultured or the study was completed. For those children in whom acquisition did not occur, the season in which the last clinical encounter was recorded was taken as the last season in which the child was at risk for acquisition.

Quasi-Poisson log-linear models with robust standard errors, modeled with the number of person-seasons of children at risk for acquisition as an offset term, were used to evaluate the seasonality of acquisition of each respiratory pathogen. Winter season served as the reference group for all analyses. Finally, we repeated the previously described analyses to evaluate whether seasonal patterns of acquisition differed within climate zones in the U.S. using the revised Köppen–Geiger climate classification [14] (Additional file 1). Results of regression analyses are reported as incidence rate ratios (IRR) and corresponding 95% confidence intervals (CI). A two-sided *P* value of 0.05 was used to determine statistical significance. All analyses were conducted using the R statistical environment (Version 3.0.2) [15].

## Results

A total of 4522 children met inclusion criteria and were included in the study population. During follow-up a total of 910 (20.1%) children acquired MRSA during 55,629 person-seasons of follow-up, 1161 (25.7%) children acquired *S*. *maltophilia* over 53,007 person-seasons, 228 (5.0%) acquired *A*. *xylosoxidans* over 60,688 person-seasons and 2148 (47.5%) of the children acquired *H. influenzae* during 42,508 person-seasons of follow-up. Among those acquiring each organism, the median age at acquisition of MRSA, *S. maltophilia*, *A. xylosoxidans* and *H. influenzae*, respectively, was 25 months (25th-75th percentiles: 14-42 months), 21 (13-35), 32 (18-48) and 20 (13-31) months. Overall, approximately 55% of individuals had a culture frequency of four per year.

Table 1 describes the demographic and clinical characteristics of the study population by acquisition status of each pathogen. Children acquiring each of the organisms were less likely to have been identified by newborn screening than those remaining infection-free. Those acquiring *S. maltophilia* and *A. xylosoxidans* were more likely to be Hispanic than those remaining free of these organisms, while the converse was true for MRSA and *H. influenzae*. The mean age of CF diagnosis was greater among children acquiring MRSA, *A. xylosoxidans* and *H. influenzae* than among those remaining free of those pathogens. Finally, patients who acquired MRSA, *S. maltophilia* and *H. influenzae* were more likely to have CF mutations resulting in minimal CFTR function.

**Table 1:** Demographic and clinical characteristics of young U.S. children with cystic fibrosis from 2003 to 2009, by pathogen acquisition status

	Respiratory Pathogen							
	MRSA		*S. maltophilia*		*A. xylosoxidans*		*H. influenzae*	
	Acquired (*n* = 910)	Negative (*n* = 3612)	Acquired (*n* = 1161)	Negative (*n* = 3361)	Acquired (*n* = 228)	Negative (*n* = 4294)	Acquired (*n* = 2148)	Negative (*n* = 2374)
Male (%)	445(49%)	1833(51%)	590(51%)	1688(50%)	98(43%)	2180(51%)	1062(49%)	1216(51%)
White (%)	825(91)	3326(92)	1065(92)	3086(92)	202(89)	3949(92)	1984(92)	2167(91)
Hispanic (%)	72(8)	427(12)*	148(13)	351(10)*	42(18)	457(11)*	208(10)	291(12)*
Identified by newborn screening (%)	280(31)	1618(45)*	423(36)	1475(44)*	73(32)	1825(43)*	806(38)	1092(46)*
Mean age at diagnosis, months (SD)	2.9(4.6)	2.4(4.3)*	2.6(4.3)	2.4(4.4)	3.5(5.3)	2.4(4.3)*	2.7(4.5)	2.3(4.2)*
ΔF508 mutation category (%)								
Homozygous	482(53)	1553(43)*	586(50)	1449(43)*	108(47)	1927(45)	996(46)	1039(44)
Heterozygous	307(34)	1437(40)	390(34)	1354(40)	77(34)	1667(39)	802(37)	942(40)
Other	79(9)	463(13)	132(11)	410(12)	32(14)	510(12)	255(12)	287(12)
CFTR functional class^a^ (%)								
Severe	660(73)	2284(63)*	817(70)	2127(63)*	150(66)	2794(65)	1442(67)	1502(63)*
Residual	61(7)	354(10)	70(6)	345(10)	12(5)	403(9)	198(9)	217(9)
Unclassified	189(21)	974(27)	274(24)	889(26)	66(29)	1097(26)	508(24)	655(28)

MRSA, methicillin-resistant *Staphylococcus aureus*; *S. maltophilia*, *Stenotrophomonas maltophilia*; *A. xylosoxidans*, *Achromobacter xylosoxidans*; *H. influenzae*, *Haemophilus influenzae*; SD, standard deviation; CFTR, cystic fibrosis transmembrane conductance regulator

* *P* < 0.05 based on Student’s *t* test with unequal variances for continuous variables or χ^2^ test for categorical variables.

^a^ CFTR functional class is defined as follows: Severe, includes children in which CFTR mutations on both alleles result in minimal CFTR function (class 1, 2, or 3), including ΔF508; Residual, at least one allele with a mutation resulting in partial CFTR function (class 4 or 5); Unclassified, both alleles with unknown functional class, or one allele with minimal CFTR function and the second with unknown functional class

Incidence of each of the pathogens, overall and by season, are presented in Table 2. *H. influenzae* incidence (50.5 per 1000 person-seasons) was highest overall, followed by *A*. *xylosoxidans* (21.9 per 1000 person-seasons), MRSA (16.4 per 1000 person-seasons), and *S*. *maltophilia* (3.8 per 1000 person-seasons). Peak incidence of MRSA (19.5 per 1000 person-seasons), *A*. *xylosoxidans* (4.9 per 1000 person-seasons), and *H. influenzae* (56.8 per 1000 person-seasons) was observed in the winter season, while *S*. *maltophilia* acquisition was highest in the summer season (23.6 per 1000 person-seasons).

**Table 2:** Overall and seasonal acquisition and incidence (per 1000 person-seasons) of methicillin-resistant *Staphylococcus aureus*, *Stenotrophomonas maltophilia*, *Achromobacter xylosoxidans*, and *Haemophilus influenzae* among young U.S. children with cystic fibrosis, 2003 to 2009

			Season							
	Overall		Winter		Spring		Summer		Autumn	
	Cases (*n*)	Incidence^a^	Cases (*n*)	Incidence^a^	Cases (*n*)	Incidence^a^	Cases (*n*)	Incidence^a^	Cases (*n*)	Incidence^a^
MRSA	910	16.4	238	19.5	207	15.4	195	13.5	270	17.4
*S. maltophilia*	1161	21.9	263	22.4	259	20.3	324	23.6	315	21.3
*A. xylosoxidans*	228	3.8	64	4.9	43	2.9	54	3.4	67	3.9
*H. influenzae*	2148	50.5	563	56.8	524	52.2	537	50.0	534	44.4

MRSA, methicillin-resistant *Staphylococcus aureus*; *S. maltophilia*, *Stenotrophomonas maltophilia*; *A. xylosoxidans*, *Achromobacter xylosoxidans*; *H. influenzae, Haemophilus influenzae*

^a^ Per 1000 person-seasons

Results of the Poisson regression evaluating the seasonality of each respiratory pathogen are presented in Table 3. Compared to winter, MRSA acquisition was less common in spring (IRR: 0.79; 95% CI: 0.65, 0.96) and summer (IRR: 0.69; 95% CI: 0.57, 0.84). *A. xylosoxidans* acquisition was also less likely in spring (IRR: 0.59; 95% CI: 0.39, 0.89). *H. influenzae* acquisition was less likely in summer (IRR: 0.88; 95% CI: 0.78, 0.99) and autumn (IRR: 0.78; 95% CI: 0.69, 0.88) seasons. No statistically significant seasonal differences were observed for *S. maltophilia* acquisition. Lastly, patterns of pathogen acquisition were similar within climate zones (Additional file 2).

**Table 3:** Results of overdispersed Poisson log-linear regression evaluating seasonal incidence of methicillin-resistant *Staphylococcus aureus*, *Stenotrophomonas maltophilia*, *Achromobacter xylosoxidans* and *Haemophilus influenzae* acquisition among young U.S. children with cystic fibrosis, 2003 to 2009

	Respiratory Pathogen			
	MRSA	*S. maltophilia*	*A. xylosoxidans*	*H. influenzae*
	IRR (95% CI)	IRR (95% CI)	IRR (95% CI)	IRR (95% CI)
Winter	1.0 Referent	1.0 Referent	1.0 Referent	1.0 Referent
Spring	0.79* (0.65, 0.96)	0.91 (0.76, 1.08)	0.59* (0.39, 0.89)	0.92 (0.81, 1.04)
Summer	0.69* (0.57, 0.84)	1.05 (0.89, 1.25)	0.69 (0.47, 1.01)	0.88* (0.78, 0.99)
Autumn	0.89 (0.75, 1.07)	0.95 (0.80, 1.12)	0.80 (0.56, 1.15)	0.78* (0.69, 0.88)

MRSA, methicillin-resistant *Staphylococcus aureus*; *S. maltophilia*, *Stenotrophomonas maltophilia*; *A. xylosoxidans*, *Achromobacter xylosoxidans*; *H. influenzae*, *Haemophilus influenzae*; IRR, incidence rate ratio; CI, confidence interval

* *P* < 0.05

## Discussion

In this study, seasonal variation was observed for rates of initial acquisition of MRSA, *A*. *xylosoxidans*, and *H. influenzae* in young U.S. children with CF, while no such variation was observed for *S. maltophilia* acquisition. Compared to winter season, MRSA acquisition was less likely in spring and summer and *A. xylosoxidans* acquisition was less likely in spring. Summer and autumn seasons were associated with decreased *H*. *influenzae* acquisition compared to the winter season. Strengths of this investigation included a large national cohort of young children with CF with frequent monitoring of respiratory microbiology.

Interestingly, we previously reported seasonal variations in *P. aeruginosa* acquisition in the same cohort of young CF patients; however, the seasonal patterns of acquisition differed, with higher incidence of *P. aeruginosa* in summer (IRR: 1.22; 95% CI: 1.07, 1.40) and autumn (IRR: 1.34; 95% CI: 1.18, 1.52) seasons compared to winter [10]. Also, while in the current analyses we observed seasonal variation in MRSA acquisition, in our prior study no seasonal variation in MSSA acquisition was observed.

The reason for these different seasonal patterns of pathogen acquisition is unclear. Seasonal factors can influence both host susceptibility and pathogen proliferation in the environment [7, 16–18]. In CF patients, *P. aeruginosa,* a ubiquitous environmental organism, is typically acquired from the environment [19]. The source of other organisms is less clear but may include the indoor or outdoor environment, clinic or hospital settings and patient-to-patient transmission. We [20] and others [21] have shown that higher ambient temperatures increase the risk of *P. aeruginosa* acquisition among CF patients, which may partially explain our observation of a higher acquisition rate in the summer. On the other hand, viral respiratory infections, more prevalent in the winter both in CF patients and in the general population, are thought to increase airway susceptibility to bacterial colonization in CF patients [22–24], which may explain the higher observed rate of acquisition of MRSA, *A. xylosoxidans* and *H. influenza* in the winter months.

MRSA colonization is associated with poorer clinical outcomes in CF patients [25, 26] and is now frequently cultured from the respiratory tracts of patients. The overall prevalence among U.S. CF patients has risen from approximately 2% in 2001 to 25% in 2012 and even in young children <2 and 2-6 years MRSA prevalence in 2012 was 11 and 18%, respectively [1]. In a multicenter study of the clonal distribution of MRSA isolates in children with CF, Champion and colleagues [27] reported that approximately 70% of all isolates were healthcare associated MRSA (HA-MRSA) strains. Further, recent studies have reported that HA-MRSA infections display a seasonal pattern in the general population, with peak rates occurring in winter months; this is in contrast to community acquired MRSA infections that peak in summer months [28, 29]. In the current investigation, MRSA incidence was highest in the winter season as would be consistent with HA-MRSA infections typically found in this population. Interestingly, we observed no seasonal variation in MSSA acquisition among young CF patients in our prior study [10]; previous investigations of seasonal variations of nasal colonization of MSSA in the general population have reported mixed results [30].

Due to rare occurrences of *S*. *maltophilia* and *A. xylosoxidans* respiratory infections in the general population, evaluation of seasonal patterns has been limited and precludes comparison to results obtained herein. To our knowledge seasonal acquisition rates of these pathogens have not previously been described in the CF population. Accordingly, *Burkholderia cepacia*, a Gram-negative bacterium ubiquitous in the natural environment, is now recognized as a clinically significant pathogen in CF patients [2]. In this study meaningful analyses of *B. cepacia* could not be performed due to the relatively few cases (*n* = 41) of *B. cepacia* in this young population. During follow-up a total of 18, 10, 10, and three cases of initial *B. cepacia* were reported in the spring, summer, autumn, and winter seasons, respectively.

The clinical significance of first isolation of bacterial pathogens from respiratory cultures in young CF patients is a topic of debate and likely varies by organism [5]. While initial *P. aeruginosa* colonization can spontaneously clear, it is likely to develop into chronic infection, which is nearly impossible to eradicate and is associated with poorer outcome and survival [31, 32]. Thus standard of care around the world is to attempt to eradicate initial *P. aeruginosa* regardless of symptoms. For other organisms, the natural history is not well described and antibiotic treatment decisions are not standardized [33, 34]. Nonetheless, a potential “window of opportunity” during seasons of higher risk may present an opportunity for more intensive monitoring or targeted interventions to minimize infection risk.

There are several limitations to the present investigation. First, the exact date of pathogen acquisition was unknown due to the non-acute, subclinical nature of these infections. In this study, culturing was generally performed at quarterly intervals that enabled a “natural” seasonal analysis of incidence patterns. Second, differentiation between subtypes of bacteria species could not be performed in this study. For example, the Registry does not contain information regarding SCCmec types or Panton Valentine leukocidin genes with which to differentiate community and healthcare-associated MRSA. Given trends in the general population, it is possible that seasonal acquisition differs by subtype. Similarly, *H. influenzae* subtypes data were not collected so seasonal patterns of these subtypes could not be evaluated. Third, our study was limited to children diagnosed with CF prior to two years of age with a maximum of six years follow-up; therefore the generalizability of results is limited to very young children. Fourth, data on viral infections was unavailable in the Registry and may play an important role in viral induced secondary bacterial infections resulting from respiratory tract damage. Finally, the majority of pathogens were identified through culturing of isolates obtained from oropharyngeal swabs. Rosenfeld and colleagues [35] previously reported a moderate sensitivity and specificity for identifying *P. aeruginosa* and MSSA by oropharyngeal swab compared to bronchoalveolar lavage in children with CF; however, the accuracy of oropharyngeal cultures for the presence of the pathogens evaluated herein in the lower respiratory tract is unknown. Therefore, results from this study more accurately reflect upper airway than lower airway pathogen acquisition.

## Conclusions

In conclusion, seasonal variations of bacterial respiratory pathogen acquisition rates were observed in a large cohort of young children with CF residing in the U.S. Future studies of factors, such as macroenvironmental variables, that may be contributing to these observed incidence patterns are needed. Similarly, the impact of global warming on both local weather patterns and extreme weather events may impact seasonal acquisition of respiratory pathogens. These findings may then contribute to our understanding of the aetiology of these infections and may provide guidance for future infection control strategies.

## Additional files

Additional file 1: Methods (DOCX 14 kb)

Additional file 2: Seasonal incidence of *methicillin-resistant Staphylococcus aureus, Stenotrophomonas maltophilia, Achromobacter xylosoxidans and Haemophilus influenzae* acquisition among children <6 years of age with cystic fibrosis in the United States from 2003 to 2009, based upon Poisson regression models with the number of individuals at risk as the offset term and winter season as the season of reference (DOCX 17 kb)

## Abbreviations

*A. xylosoxidans*: *Achromobacter xylosoxidans*
*B. cepacia*: *Burkholderia cepacia*
CF: Cystic fibrosis
CFTR: Cystic fibrosis transmembrane conductance regulator
CI: Confidence interval
*H. influenzae*: *Haemophilus influenzae*
IRR: Incidence rate ratio
MRSA: Methicillin-resistant *Staphylococcus aureus*
MSSA: Methicillin-susceptible Staphylococcus aureus
*P. aeruginosa*: *Pseudomonas aeruginosa*
*S. maltophilia*: *Stenotrophomonas maltophilia*
SD: Standard deviation
