# Supplementary material for: Seasonality of acquisition of respiratory bacterial pathogens in young children with cystic fibrosis
Source: BMC Infect Dis. 2017 Jun 9;17:411. doi: 10.1186/s12879-017-2511-9 (PMC5466772; doi:10.1186/s12879-017-2511-9)
Supplement: Supplementary file 1 — Methods (DOCX 14 kb) [file 12879_2017_2511_MOESM1_ESM.docx]

Additional file 1

Methods

As seasonal conditions vary in the U.S. based on geographic location we evaluated whether seasonal differences in pathogen acquisition varied within climate zones in the U.S. using the revised Köppen–Geiger Climate classification. This classification scheme is comprised of five broad climate regions (Tropical, Dry, Temperate, Continental and Polar) which are defined based on meteorological variables; in the continental U.S. all but one (Polar) of these regions are represented.

Individual-level residential zip code data from the CFF Registry was used to spatially reference all children to their corresponding zip code centroid using the residential zip code in the year in which the pathogen was acquired, for those children who acquired the pathogen during follow-up, or the year of the last clinical visit recorded in the Registry (for those remaining pathogen-free). We then determined the child’s climate classification by linking each individual’s zip code centroid to the revised Köppen–Geiger climate classification using Arc GIS version 10.1 (ESRI, Redlands, CA, USA). Due to the minimal number of patients residing in the tropical zone (n = 37), seasonal variability in this zone was not evaluated.
