# Supplementary material for: Seasonality of acquisition of respiratory bacterial pathogens in young children with cystic fibrosis
Source: BMC Infect Dis. 2017 Jun 9;17:411. doi: 10.1186/s12879-017-2511-9 (PMC5466772; doi:10.1186/s12879-017-2511-9)
Supplement: Supplementary file 2 — Seasonal incidence of methicillin-resistant Staphylococcus aureus, Stenotrophomonas maltophilia, Achromobacter xylosoxidans and Haemophilus influenzae acquisition among children <6 years of age with cystic fibrosis in the United States from 2003 to 2009, based upon Poisson regression models with the number of individuals at risk as the offset term and winter season as the season of reference (DOCX 17 kb) [file 12879_2017_2511_MOESM2_ESM.docx]

Additional file 2

Seasonal incidence of *methicillin-resistant Staphylococcus aureus, Stenotrophomonas maltophilia, Achromobacter xylosoxidans and Haemophilus influenzae* acquisition among children <6 years of age with cystic fibrosis in the United States from 2003-2009, based upon Poisson regression models with the number of individuals at risk as the offset term and winter season as the season of reference.

|  | **Climate Zone** | | |
| --- | --- | --- | --- |
|  | Dry | Temperate | Continental |
|  | IRR  (95% CI) | IRR  (95% CI) | IRR  (95% CI) |
| MRSA |  |  |  |
| Winter | 1.0  Referent | 1.0  Referent | 1.0  Referent |
| Spring | 0.34*  (0.13-0.90) | 0.85  (0.65-1.11) | 0.78  (0.58-1.04) |
| Summer | 0.58  (0.26-1.28) | 0.71*  (0.54-0.93) | 0.70*  (0.52-0.94) |
| Autumn | 0.79  (0.38-1.61) | 0.91  (0.71-1.17) | 0.88  (0.67-1.16) |
| *S. maltophilia* |  |  |  |
| Winter | 1.0  Referent | 1.0  Referent | 1.0  Referent |
| Spring | 0.85  (0.39-1.86) | 0.84  (0.65-1.10) | 0.97  (0.76-1.24) |
| Summer | 1.10  (0.54-2.72) | 0.95  (0.74-1.22) | 1.13  (0.90-1.43) |
| Autumn | 1.27  (0.64-2.53) | 0.90  (0.70-1.16) | 1.01  (0.80-1.28) |
| *A. xylosoxidans*^†^ |  |  |  |
| Winter | 1.0  Referent | 1.0  Referent | 1.0  Referent |
| Spring | - | 0.68  (0.36-1.29) | 0.58  (0.33-1.00) |
| Summer | - | 0.78  (0.43-1.42) | 0.69  (0.41-1.15) |
| Autumn | - | 0.90  (0.51-1.60) | 0.93  (0.59-1.49) |
| *H. influenzae* |  |  |  |
| Winter | 1.0  Referent | 1.0  Referent | 1.0  Referent |
| Spring | 0.75  (0.48-1.17) | 1.00  (0.83-1.21) | 0.90  (0.76-1.07) |
| Summer | 0.67  (0.43-1.05) | 0.86  (0.71-1.04) | 0.94  (0.80-1.12) |
| Autumn | 0.83  (0.55-1.25) | 0.71*  (0.58-0.86) | 0.86  (0.73-1.02) |

MRSA, methicillin-resistant *Staphylococcus aureus*; *S. maltophilia*, *Stenotrophomonas maltophilia*; *A.* *xylosoxidans*, *Achromobacter xylosoxidans*; *H. influenzae*, *Haemophilus* *influenzae*; IRR, incidence rate ratio; CI, confidence interval

^*^ *P* <0.05

^†^ Results of regression models are not reported due to the minimal number of *A. xylosoxidans* acquisition in the Dry climate zone.
